# BCG-Mediated Protection against *Mycobacterium ulcerans* Infection in the Mouse

**DOI:** 10.1371/journal.pntd.0000985

**Published:** 2011-03-15

**Authors:** Paul J. Converse, Deepak V. Almeida, Eric L. Nuermberger, Jacques H. Grosset

**Affiliations:** Johns Hopkins University Center for Tuberculosis Research, Baltimore, Maryland, United States of America; Yale University School of Medicine, United States of America

## Abstract

**Background:**

Vaccination with *Mycobacterium bovis* bacille Calmette-Guérin (BCG) is widely used to reduce the risk of childhood tuberculosis and has been reported to have efficacy against two other mycobacterial diseases, leprosy and Buruli ulcer caused by *M. ulcerans* (*Mu*). Studies in experimental models have also shown some efficacy against infection caused by *Mu*. In mice, most studies use the C57BL/6 strain that is known to develop good cell-mediated protective immunity. We hypothesized that there may be differences in vaccination efficacy between C57BL/6 and the less resistant BALB/c strain.

**Methods:**

We evaluated BCG vaccine efficacy against challenge with ∼3×10^5^ *M. ulcerans* in the right hind footpad using three strains: initially, the Australian type strain, designated Mu1617, then, a Malaysian strain, Mu1615, and a recent Ghanaian isolate, Mu1059. The latter two strains both produce mycolactone while the Australian strain has lost that capacity. CFU of both BCG and *Mu* and splenocyte cytokine production were determined at intervals after infection. Time to footpad swelling was assessed weekly.

**Principal Findings:**

BCG injection induced visible scars in 95.5% of BALB/c mice but only 43.4% of C57BL/6 mice. BCG persisted at higher levels in spleens of BALB/c than C57BL/6 mice. Vaccination delayed swelling and reduced *Mu* CFU in BALB/c mice, regardless of challenge strain. However, vaccination was only protective against Mu1615 and Mu1617 in C57BL/6 mice. Possible correlates of the better protection of BALB/c mice included 1) the near universal development of BCG scars in these mice compared to less frequent and smaller scars observed in C57BL/6 mice and 2) the induction of sustained cytokine, e.g., IL17, production as detected in the spleens of BALB/c mice whereas cytokine production was significantly reduced, e.g., IL17, or transient, e.g., Ifnγ, in the spleens of C57BL/6 mice.

**Conclusions:**

The efficacy of BCG against *M. ulcerans*, in particular, and possibly mycobacteria in general, may vary due to differences in both host and pathogen.

## Author Summary

Vaccination with *Mycobacterium bovis* bacille Calmette-Guérin (BCG) is used to reduce the risk of childhood tuberculosis and is reported to have efficacy against two other diseases also caused by mycobacteria, leprosy and Buruli ulcer caused by *M. ulcerans*. We hypothesized that there may be differences in the effectiveness of BCG vaccination in different mouse strains. We vaccinated two mouse strains with BCG eight weeks before infection with three different strains of *M. ulcerans*. Two of the bacterial strains make a toxin that is critical for Buruli ulcer disease and the third does not. We observed the progression of disease in vaccinated and mock-vaccinated mice and also evaluated the immune response of the mice. We found that the BALB/c mice respond to BCG vaccination with prominent scars, a vigorous immune response, and delayed or no manifestations of *M. ulcerans* infection. C57BL/6 mice, on the other hand, usually do not have vaccination scars, make a relatively short-lived and/or weaker immune response, and all show disease at the site of *M. ulcerans* infection. We conclude that the efficacy of BCG against *M. ulcerans*, and possibly other diseases, depends on the nature of the host and of the infecting strain of the bacteria.

## Introduction

BCG vaccination is widely practiced around the world, primarily to protect against tuberculosis. BCG is a safe vaccine but its efficacy against tuberculosis varies by geographical region and possibly by BCG strain due to mutations related to culturing practices in multiple laboratories for many decades. The current consensus is that it protects against disseminated tuberculosis in young children but that it has limited value in protecting against adult pulmonary tuberculosis, perhaps affording 50% protection at best [1]. On the other hand, large trials [2] have shown that even where BCG has no discernible benefit against tuberculosis, it does protect against leprosy, a disease caused by another mycobacterium, *M. leprae*. Against yet another mycobacterial disease, known as *Mycobacterium ulcerans* disease or Buruli Ulcer (BU), retrospective and prospective studies have found that BCG vaccination appears to have protective efficacy for only up to 6 months but there may be longer term protection against severe forms of BU, such as osteomyelitis [3], [4], [5], [6], [7]. A case report indicated that the Th1 type immunity following BCG vaccination changed to a Th2 type after the onset of BU [8], [9]. Our preliminary investigation of a toxin-negative *Mu* strain and studies by others suggested differences in host response between C57BL/6 and BALB/c mice [10], [11], [12], and therefore, studies in mice might help identify the timing and nature of the switch and allow testing of alternative ways to maintain protective immunity.

*Mycobacterium ulcerans* disease was first described in the medical literature in 1948 in Australian patients [13]. The disease still occurs there, primarily in coastal areas visited by vacationers. In contrast, many more cases have been documented to occur in Africa, initially in the Congo [14] and Uganda [15], and then, increasingly in West Africa where it primarily affects impoverished people in rural riverine and swampy areas [16], [17], [18]. The exact mode of transmission is controversial. Bug bites are frequently but not universally recalled. *M. ulcerans* grows slowly at ∼30–32 °C. It was the first mycobacterium shown to produce a toxin, an immunosuppressive macrolide, named mycolactone [19], [20], [21]. Toxin-producing colonies have a yellowish color. The toxin is encoded by the *pksA* gene, located on a giant plasmid [22], [23], [24]. The toxin destroys subcutaneous fat cells, apparently by both apoptotic and necrotic mechanisms [25], [26], [27]. Both guinea pigs and mice have been used to model the disease and study the organism [21], [28], [29], [30]. In mice injected in the hind footpad, there is gradual, infection-dose-dependent swelling that becomes severe before the onset of ulceration and, if allowed, may progress to foot and limb loss and death [31], [32].

Here, we vaccinated BALB/c and C57BL/6 mice with BCG (Pasteur) and, after 8 weeks, challenged vaccinated and unvaccinated mice with either *M. ulcerans* 1059 (Mu1059), a recent clinical isolate from Ghana, or with Mu1615, a strain originally isolated from Malaysia in the 1960s. Both strains produce mycolactone and both cause a gradually severe swelling in mouse footpads in unvaccinated mice.

## Materials and Methods

### Bacteria

*M. ulcerans* 1059 (Mu1059, a recent clinical isolate from Ghana) and *M. ulcerans* 1615 (Mu1615, an isolate originally obtained from a patient in Malaysia in the 1960s, [33] were kindly provided by Dr. Pamela Small, University of Tennessee. *M. ulcerans* 1617 (Mu1617, the type strain isolated from a patient in Australia in the 1940s, [13]) was obtained from the American Type Culture Collection (ATCC, Manassas, VA). Thin layer chromatographic analysis and cytotoxicity assays of ethanolic extracts showed that Mu1059 and Mu1615, but not Mu1617, produce mycolactone and kill macrophages and fibroblasts ([34], and see Fig. S3). All three strains were passaged in mouse footpads before use in these studies. The bacilli were harvested from swollen footpads at the grade 3 level, i.e., swelling with inflammation of the footpad and leg [35].

### BCG Vaccination

Female BALB/c and C57BL/6 mice, aged 4–6 weeks, obtained from Charles River (Wilmington, MA), were vaccinated subcutaneously with 5.8×10^4^ CFU of *M. bovis* BCG, Pasteur strain, in 0.2 ml or with the diluent (Middlebrook 7H9, also 0.2 ml) as a sham manipulation, 8 weeks before challenge with *M. ulcerans*.

### Challenge and CFU analysis

Mice were inoculated in the right hind footpad with approximately 3×10^5^ in 0.03 ml of Mu1059, Mu1615, or Mu1617. At different time points after challenge, mice were sacrificed and footpad tissue was harvested, minced with fine scissors [36], suspended in 2.0 ml PBS, serially diluted, and plated for CFU analysis on Middlebrook selective 7H11 plates (Becton-Dickinson, Sparks, MD). Mice were evaluated for footpad swelling weekly using an established scoring system [35] with grade 1 showing footpad swelling, grade 2 swelling with inflammation, and grade 3 swelling and inflammation of the entire foot [32]. Time to grade 1 swelling was assessed by Kaplan-Meier analysis. All animal procedures were conducted according to relevant national and international guidelines. The study was conducted adhering to the Johns Hopkins University guidelines for animal husbandry and was approved by the Johns Hopkins Animal Care and Use Committee, protocols MO08M240 and MO05M226.

### Splenocyte assays

After BCG or sham vaccination, spleens were harvested from mice, placed in 2.5 ml RPMI 1640 (Mediatech, Herndon, VA) and passed through a 70 µm cell strainer (BD Falcon 352350) into a centrifuge tube. From the suspension, 120 µl was added to 1.88 ml of RPMI containing 5% fetal bovine serum and 1% penicillin (100 U/ml) and streptomycin (100 µg/ml). From this suspension 100 µl was added to triplicate wells of a 96 well plate (Costar 3595, Corning, NY) containing 10 µl of culture filtrate protein of *Mtb* H37Rv (CFP, [10 µg/ml]) or *Mtb* antigen 85 (Ag85), also at 10 µg/ml, both supplied by Colorado State University TB Vaccine Testing and Research Materials Contract (NIH-NIAID N01-AI-40091), or with Concanavalin A (ConA, Sigma, [2 µg/ml]). The cells were incubated at 37 °C for 48 hours before harvesting, pooling, and freezing at −70 °C of triplicate 50 µl supernatants. The remainder of the suspension was used to enumerate BCG CFU.

### Cytokine assays

A 23-plex Luminex assay (Biorad, Hercules, CA) was used to detect 4 Th1 (IL2, IL12b/p40, IL12p70, and Ifnγ), 4 Th2 (IL4, IL5, IL10, and IL13), and 4 proinflammatory cytokines (IL1α, IL1β, IL6, and Tnfα) as well as IL17, IL9, 6 chemokines (Cxcl1, Ccl2, Ccl3, Ccl4, Ccl5, and Ccl11), and 3 colony stimulating factors (IL3, Csf2, and Csf3). Using aliquots from the same supernatants, Tgfβ was detected using Milliplex beads (Millipore, Billerica, MA).

### Statistical analysis

Comparisons were made by the log-rank test for time-to-grade 1 swelling and by 2-way ANOVA for log_10_ transformed CFU counts and cytokine levels.

## Results

### BCG vaccination produces scars more frequently in BALB/c than in C57BL/6 mice

**Figure 1:**
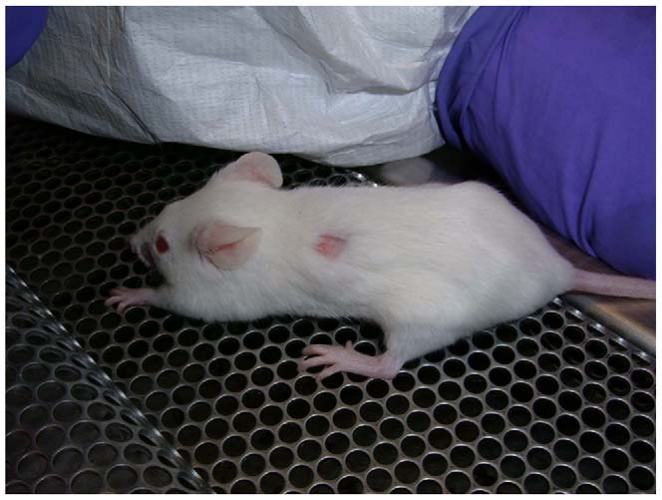
After subcutaneous vaccination with BCG-Pasteur, BALB/c mice consistently have visible scars. Scars were less frequently seen in C57BL/6 mice and tended to be smaller. The pictured mouse was vaccinated 10 weeks previously and had been challenged with *M. ulcerans* 2 weeks previously in the right hind footpad.

Nearly all BALB/c mice developed visible scars at the site of vaccination in the flank (Fig. 1) but such scars occurred less frequently and were smaller in C57BL/6 mice. This observation was assessed and noted for each mouse 13 weeks after vaccination and 5 weeks after *M. ulcerans* challenge. In all, 95.5% of BALB/c mice but only 43.4% of C57BL/6 mice had scars after BCG vaccination. No sham-vaccinated mice had scars.

### After subcutaneous vaccination, BCG multiplies in spleen and survives longer in BALB/c mice

**Figure 2:**
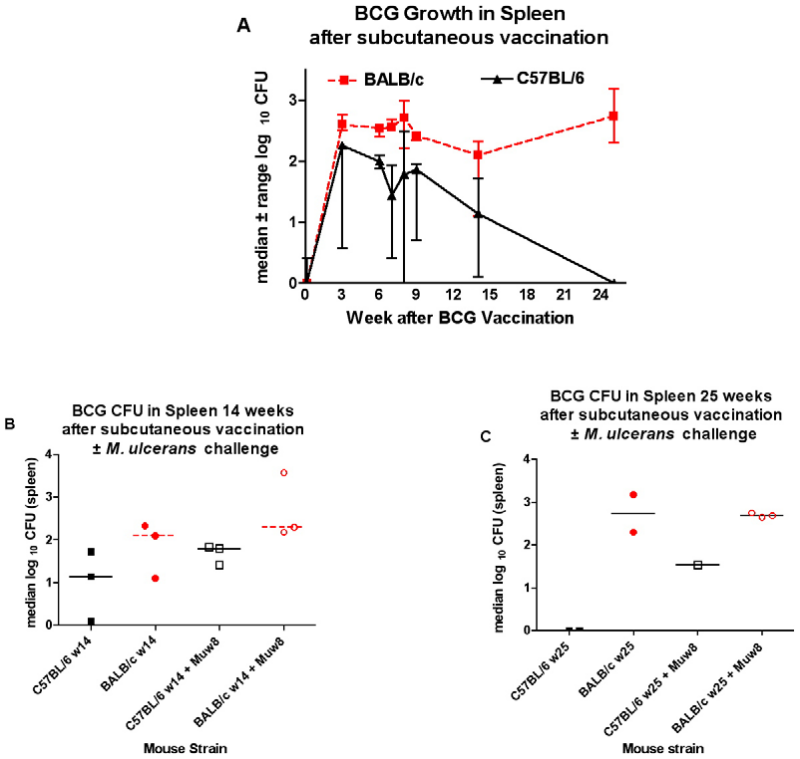
After subcutaneous vaccination BCG persists in spleen longer in BALB/c than in C57BL/6 mice. Variability is greater in the C57BL/6 mice. At week 8, mice were challenged with *M. ulcerans*. By week 25, no BCG colonies could be detected in the C57BL/6 mice (A, C). (N = 3, except at week 25 where N = 2; one of two comparable experiments shown). At week 14 (B), BCG CFU appeared to be higher in C57BL/6 mice challenged with *M. ulcerans*. (C) The one C57BL/6 mouse available at week 25 after vaccination and 17 weeks after *M. ulcerans* challenge did have detectable BCG in the spleen.

C57BL/6 mice are considered to be more resistant than BALB/c mice to intracellular infections with mycobacteria or leishmania species [37], [38]. Consistent with these observations we found that after subcutaneous *M. bovis* BCG vaccination, BALB/c mice have higher levels of BCG detectable in the spleen than do C57BL/6 mice (Fig. 2 A, B, C). In addition, the levels persisted longer in BALB/c mice. The number of BCG CFU in the spleen tended to be higher in *M. ulcerans*-challenged C57BL/6 compared to unchallenged mice (Fig. 2 B and C) whereas there was no difference between challenged and unchallenged BALB/c mice.

### *M. ulcerans* infection is established at comparable levels in BALB/c and C57BL/6 mice with all 3 bacterial strains

**Figure 3:**
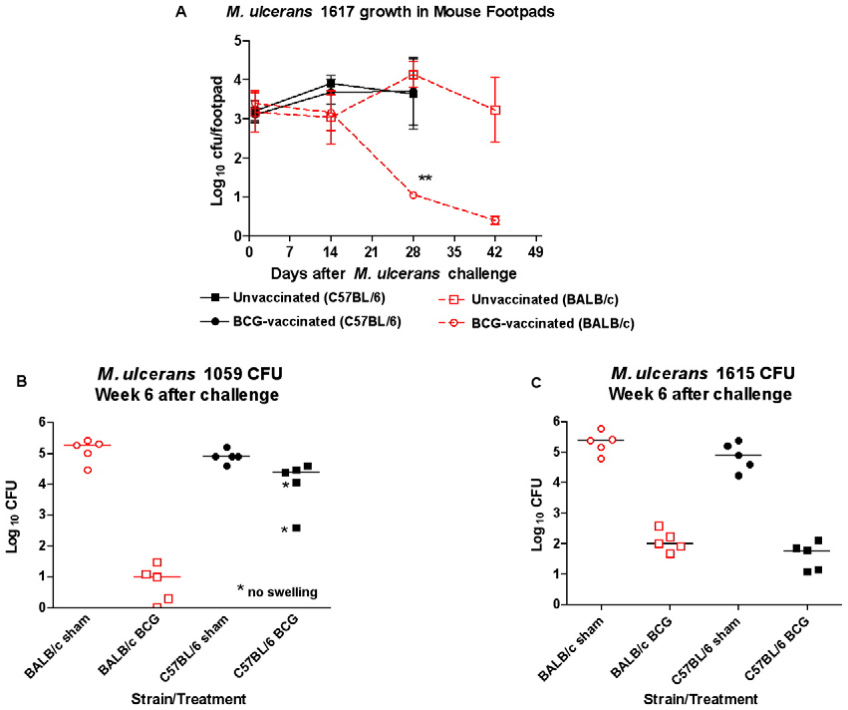
*M. ulcerans* infection is established comparably in BALB/c and C57BL/6 mice. A) CFU counts of the Mu1617 strain of *M. ulcerans* are comparable between C57BL/6 and BALB/c mice and with the mycolactone-producing Ghanaian strain, Mu1059 (not shown) regardless of BCG vaccination status. *M. ulcerans* CFU at day 28 are significantly lower in vaccinated BALB/c mice (**, p<0.01). B) and C): Mice that show a protective response to BCG vaccination have reduced *M. ulcerans* CFU after the onset of swelling (week 5 or 6) in unprotected, i.e., unvaccinated, mice.

Mice were inoculated with freshly isolated suspensions containing ∼10 AFB/high-power microscopic field or 2.4–3×10^5^ *M. ulcerans* in a volume of ∼0.03 ml in the right hind footpad, resulting in an implantation of ∼3.1–3.2 log_10_ of the Australian type strain, Mu1617 on day 1 (Fig. 3A). As reported by others (e.g., [39]) there is a lag phase with little or no increase in CFU during the first 2 weeks after challenge, followed by a logarithmic increase accompanied by footpad swelling by week 5 to 6 when the CFU counts reach 10^5^ or higher. For example at week 2 after infection, we detected from ∼3–3.5 log_10_ *M. ulcerans* CFU in the mouse footpads infected with Mu1059 or Mu1615, independent of BCG vaccination status (data not shown).

### BCG vaccination prevents multiplication of *M. ulcerans* better in BALB/c than in C57BL/6 mice

At week 6, after the onset of swelling in the sham-vaccinated mice, marked differences in *M. ulcerans* CFU counts were observed between BCG-vaccinated and unvaccinated (i.e., sham) BALB/c mice regardless of the infecting *M. ulcerans* strain (Mu1617, p<0.01, Mu 1059, p<0.001, or Mu1615, p<0.001, Figure 3). In C57BL/6 mice BCG vaccination did not result in a reduction in Mu1617 (Fig. 3A) or Mu1059 CFU (Fig. 3B) but did lead to a significant (p<0.01) reduction in *M. ulcerans* 1615 CFU (Fig. 3C) in the footpads of mice.

### BCG vaccination delays time to footpad swelling in a *M. ulcerans* strain- and mouse strain-dependent manner

**Figure 4:**
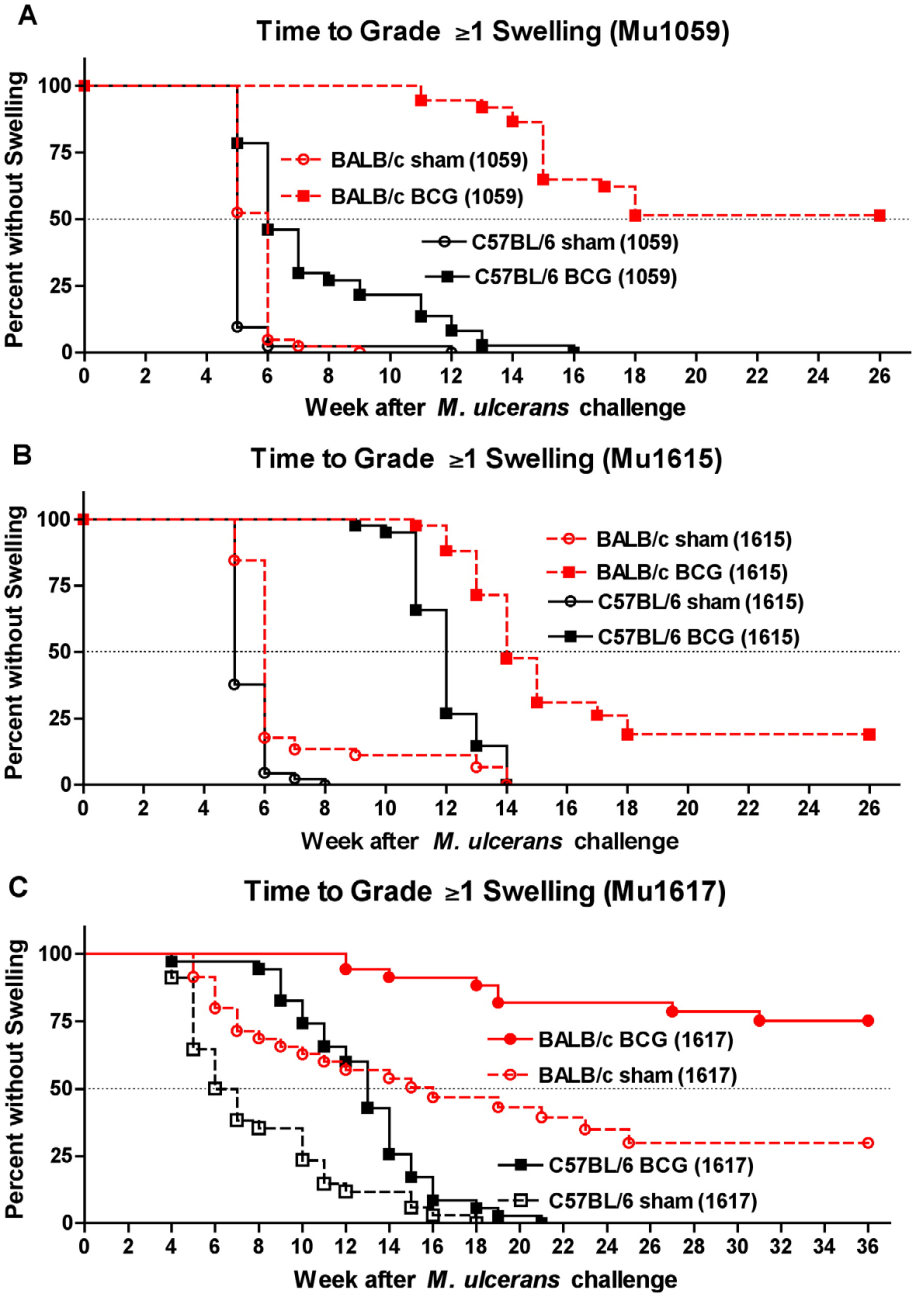
Swelling of mouse footpads after *M. ulcerans* infection is significantly delayed in BALB/c mice and, to a more variable extent, in C57BL/6 mice after BCG vaccination. BCG-mediated protection against swelling was greatest in BALB/c mice, regardless of *M. ulcerans* strain. Significant protection was also observed in C57BL/6 mice against the toxin-producing Malaysian strain, Mu1615 (B), and the non-toxin-producing Australian type strain, Mu1617 (C), but negligible protection was observed with the Ghanaian strain, Mu1059 (A). It is noteworthy that swelling occurred after infection with the non-toxin-producing Mu1617 strain (C) but that the onset of swelling was delayed even in unvaccinated BALB/c mice.

In unvaccinated BALB/c and C57BL/6 mice, the median time to grade 1 or higher footpad swelling was ∼5–6 weeks following infection with either of the toxin-producing strains (Fig. 4A and 4B) whereas it was 6.5 weeks in C57BL/6 mice and 16 weeks in BALB/c mice infected with the non-toxin-producing Mu1617 strain (Fig. 4C). All unvaccinated BALB/c and C57BL/6 mice developed footpad swelling except for 37% of BALB/c mice challenged with the Mu1617 strain.

In BCG vaccinated BALB/c mice, the median time to swelling was >26 weeks in mice challenged with the Mu1059 Ghanaian strain (Fig. 4A) and 14 weeks with the Mu1615 Malaysian strain (Fig. 4B). Only 23% of vaccinated BALB/c mice developed swelling by week 36 after infection with the toxin free, Mu1617 Australian strain (Fig. 4C). Remarkably, approximately 25%, 50, or 75% of, vaccinated BALB/c mice infected with Mu1615, Mu1059, and Mu1617, respectively, never developed swelling. In addition, some appeared to self-heal with a regression of the swelling.

In contrast, the effect of BCG vaccination on median time to swelling in C57BL/6 mice varied from a delay of only 1 week in mice challenged with the Mu1059 strain (Fig. 4A) to a delay of 7 weeks in mice challenged with the Mu1615 and Mu1617 strains (Fig. 4B and 4C). All vaccinated C57BL/6 mice eventually developed swelling regardless of the challenge strain. Taken together, these results suggest that vaccine efficacy varies according to both the mouse strain and the *M. ulcerans* strain that were tested.

### BCG vaccine efficacy correlates with BCG scar formation and with sustained cytokine production in response to mycobacterial antigens

At different times after vaccination, splenocytes were assessed for the ability to produce cytokines after restimulation with CFP or Ag85 of *Mtb* as well as Concanavalin A. The responses to Ag85 correlated very well with those to CFP. Because we tested more time points with CFP, we report, for the sake of simplicity, only those results. Similar results for cytokine production were observed after challenge with either Mu1615 or Mu1059 and, therefore, those data are combined.

#### Proinflammatory cytokines

**Figure 5:**
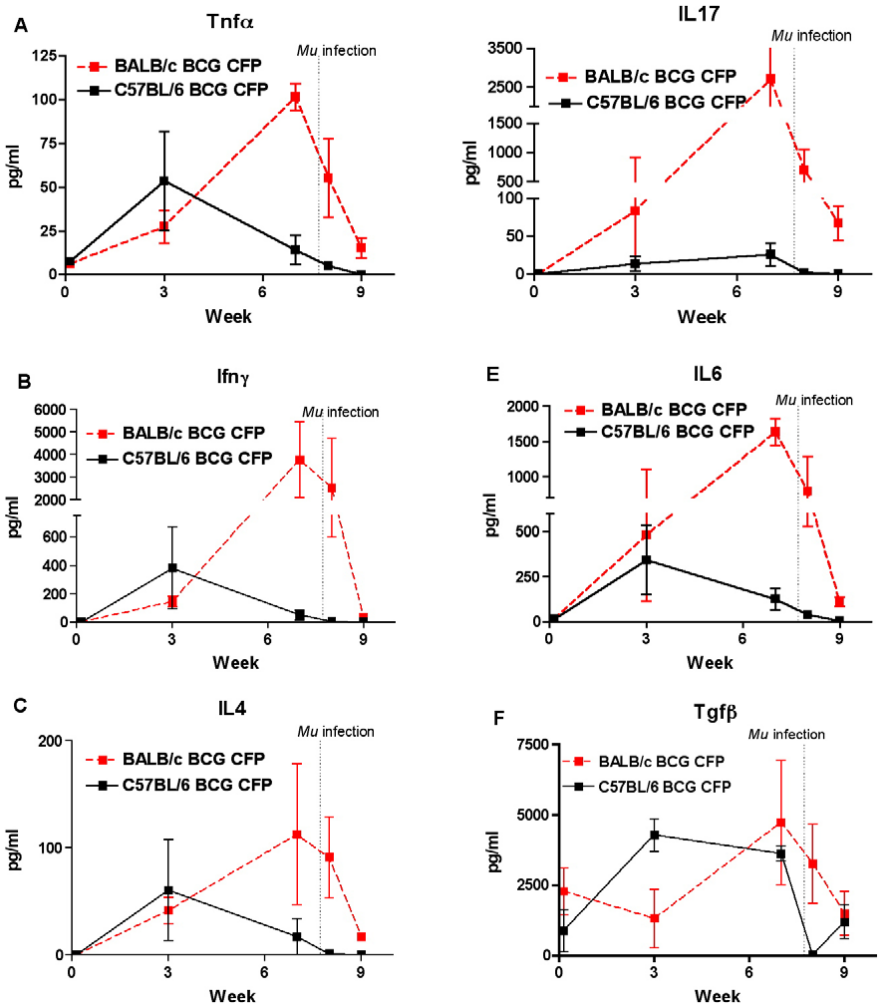
Cytokine production in spleens of BCG-vaccinated C57BL/6 and BALB/c mice before and after challenge with *M. ulcerans* strains Mu1615 and Mu1059. Representative proinflammatory (A), Th1 (B), and Th2 (C) cytokines are shown. IL17 production is shown in Panel D. IL6, required for the generation of Th17 cells, is shown in panel E. Tgfβ, required for the generation of both Th17 and regulatory T (Treg) cells, production is shown in panel F. Graphs of other cytokines (Fig. S1) and also chemokines (Fig. S2) are available in Supplementary Materials.

The proinflammatory cytokines IL1α and IL1β were produced at identical levels and rates over the first 3 weeks after BCG vaccination in both mouse strains. Thereafter, production increased markedly in BALB/c but decreased in C57BL/6 mice at week 7. Production declined after challenge with *M. ulcerans* in both mouse strains when tested 8 weeks after BCG vaccination and one week after challenge. Two weeks after challenge, IL1 could not be detected in supernatants of C57BL/6 splenocytes stimulated with CFP (Fig. S1). The same pattern was observed for Tnfα (Fig. 5 A) and IL6 (Fig. 5E), except that the former was somewhat higher in C57BL/6 mice and the latter was higher in BALB/c mice. The differences at 3 weeks were not statistically significant.

#### Th1 cytokines

The same kinetics of expression was observed for Th1 cytokines Ifnγ, IL2, IL12β, and IL12 p70 (Fig. S1). The peak at week 3 was higher for Ifnγ (Fig. 5B) and IL12β in C57BL/6 than in BALB/c mice; however, the differences were not statistically significant by 2-way ANOVA. IL12p70 was undetectable in C57BL/6 mice even before *M. ulcerans* challenge. Both Ifnγ and IL12p70 were undetectable 2 weeks after *M. ulcerans* challenge in both mouse strains.

#### Th2 cytokines

Again the same kinetics pattern of sustained cytokine production in BALB/c mice but a peak in production at week 3 in C57BL/6 mice was observed for the Th2 cytokines IL4 (Fig. 5C), IL5, IL10, and IL13 (Fig. S1).

#### Th17 cytokines

The pattern and the level of IL17 (Fig. 5D) production in response to CFP were markedly different between the two mouse strains. At week 3 the mean IL17 concentrations, in pg/ml, were 84±41 (range, 40–166) for BALB/c mice but 13±6 (range, 3–21) for C57BL/6 mice. At week 7 the concentrations were 2703±487 for BALB/c mice and 26±9 for C57BL/6 mice. After *M. ulcerans* challenge, IL17 was almost undetectable one week later in C57BL/6 mice whereas in BALB/c mice levels only declined to 705±203 and 67±13 in the two weeks after challenge. Among the factors required for the generation of Th17cells are IL6 and Tgfβ. As noted above, IL6 (Fig. 5E) levels were initially somewhat, though not significantly, higher in BALB/c than in C57BL/6 mice and, as with other cytokines, continued to increase in the BALB/c mice while declining in the C57BL/6 mice. The pattern for Tgfβ (Fig. 5F) was different from the other cytokines. Values were 2.5-fold higher at day 1, 3.2-fold lower at 3 weeks but again slightly higher at 7 weeks in BALB/c mice compared to C57BL/6 mice, although the differences were not statistically different. Tgfβ values also remained higher after *M. ulcerans* challenge in BALB/c mice but dropped precipitously in C57BL/6 mice. The results for Tgfβ could be consistent with the cytokine helping to drive, through the Rorγt transcription factor, a Th17 response in BALB/c mice while inducing a regulatory T cell (Treg) response, through the Foxp3 transcription factor, in C57BL/6 mice in which cytokine production was consistently down modulated after week 3. Taken together, the data suggest that the strong IL17 response in BALB/c mice compared to C57BL/6 mice correlates with BCG vaccine-mediated protection against *M. ulcerans*-induced footpad swelling and the reduction in *M. ulcerans* CFU detectable in BALB/c footpads.

Data for all 12 cytokines tested are presented in Fig. S1 and for 10 chemokines in Fig. S2.

## Discussion

BCG vaccination protects BALB/c mice better than C57BL/6 mice from the consequences of *M. ulcerans* infection. In the case of infection with the Mu1059 isolate from Ghana, C57BL/6 mice were essentially not protected at all whereas most BALB/c remained swelling free. In general, BALB/c mice make a stronger and more sustained cytokine response than do C57BL/6 mice. The most salient difference in cytokine production between the two mouse strains was the IL17 response. IL17 is known to be associated with protection against extracellular fungi and bacteria [40].

Others have also observed differential production of IL17 between C57BL/6 and BALB/c mouse strains [38]. In contrast to our findings of an association between high levels of IL17 and BCG-induced protection from *M. ulcerans* disease, Lopez Kostka found that BALB/c mice produced “excessive” levels of IL17, as well as Th2 cytokines, and are more susceptible to cutaneous leishmaniasis after infection with *L. major* [38]. In the leishmania model, C57BL/6 mice have a strong Th1 response but produce little IL17. In this model, killing of the organism occurs following Ifnγ production and macrophage activation with elaboration of nitric oxide. BCG, on the other hand, induces granuloma formation that may help contain mycobacteria. Very recently, Okamoto Yoshida *et al.* reported that mice lacking IL17, in a C57BL/6 background, fail to produce granulomas after pulmonary BCG infection [41]. Whether subcutaneous BCG vaccination promotes granuloma formation in footpads and protection against *M. ulcerans* infection requires further investigation.

Our findings in BALB/c mice are consistent with those of Coutanceau *et al*. [10] who also observed a >3 log_10_ reduction in Mu1615 CFU at the site of infection in mice vaccinated subcutaneously with BCG Pasteur (Fig. 3C). The results here extend the data to C57BL/6 mice in which there was also a 3.26 log_10_ reduction in Mu1615 CFU at 6 weeks after challenge. An earlier study [12] found that in intravenously BCG-vaccinated C57BL/6 mice challenged with *M. ulcerans* strain 5150 from the Congo, there was only a 1.35–1.85 log_10_ reduction in the footpad CFU counts 7 weeks after challenge. We likewise saw a reduction of only 0.88 log_10_ CFU in vaccinated C57BL/6 mice challenged with the Ghanaian Mu1059 strain. However, vaccinated BALB/c mice challenged with Mu1059 showed a 4.32 log_10_ reduction in CFU. These data strongly support the idea that there are host differences in the ability to be protected by BCG from *M. ulcerans* infection as well as marked differences in the protection conferred against different strains of *M. ulcerans*.

In addition to counting *M. ulcerans* CFU, we assessed the ability of BCG vaccination to prevent footpad swelling, a clinically observable consequence of *M. ulcerans* infection in mice. BCG vaccination delayed the onset of footpad swelling in both BALB/c and C57BL/6 mice. However, the delay was greater in BALB/c mice, regardless of the challenge strain and C57BL/6 mice showed only a 1-week delay in swelling when challenged with the Ghanaian Mu1059 strain. These findings support the differences found in CFU and also tend to confirm the difference in host susceptibility to *M. ulcerans* noted parenthetically by others [10].

Differential susceptibility to mycobacterial infection in mice has been the subject of numerous studies. BALB/c and C57BL/6 mice have very similar survival rates after aerosol infection with *M. tuberculosis*. Both strains are markedly resistant compared to CBA, DBA/2, C3H, and 129/SvJ when challenged by the intravenous or aerosol routes. Interestingly, the distinction was overcome by increasing the i.v. challenge dose [42]. This study was followed up by evaluating the ability of BCG vaccination to protect BALB/c and DBA/2 (both having the same MHC type) against an intravenous challenge with *M. tuberculosis.* For both strains there was a ∼10-fold reduction in the number of CFU in the lung 80 days after challenge. However, at this time, there was also a 100-fold difference in the number of CFU in the lungs of the immunized BALB/c and DBA/2 mice. In addition, the DBA/2 mice also had extensive necrotic lesions whereas the BALB/c lesions were more compact and epithelioid like [43]. Similar findings were obtained when comparing vaccinated C57BL/6 mice and the susceptible strains, DBA/2 and CBA/J [44]. Other studies have linked differential susceptibility to matrix metalloproteinases such as Mmp9 [45]. Hence, the finding of a difference in the susceptibility of different mouse strains to mycobacteria is not novel but the difference in susceptibility of BALB/c and C57BL/6 mice, both resistant to *M. tuberculosis*, to *M. ulcerans* has not been shown before nor, to our knowledge, has the difference in the ability of BCG vaccination to protect these different mouse strains been examined before.

The importance of IL17 may be due to the fact that the intracellular phase is relatively brief after *M. ulcerans* infection due to toxin-mediated killing of phagocytic cells whereas, in mice, *M. tuberculosis* infection remains intracellular throughout the course of infection (unpublished observations and [46]). Studies in progress indicate that at week 2 after infection with a mycolactone-producing strain, Mu1615, the organisms are still largely intracellular. By week 3, the infection is predominantly extracellular in BALB/c mice, presumably due to the destruction of phagocytes by mycolactone. BCG vaccination may promote IL17 production, particularly in this mouse strain, and enable resistance against extracellular organisms. In contrast, C57BL/6 mice, infected with Mu1617, which does not produce mycolactone, have abundant organisms that appear to be intracellular, even at 4 weeks after infection.

The results of this study suggest that vaccination with BCG may protect some hosts more effectively than others against *M. ulcerans* infection or disease. In addition, the protection may depend on the strain of *M. ulcerans* prevalent in a given community. While the benefit of BCG vaccination may be variable, we also found no evidence of vaccination leading to exacerbated disease in this model.

## Supporting Information

Figure S1 Proinflammatory, Th1, and Th2 cytokine production after BCG vaccination before and after *M. ulcerans* challenge.
(0.04 MB DOC)

Figure S2 Chemokine production after BCG vaccination before and after *M. ulcerans* challenge.
(0.03 MB DOC)

Figure S3 Thin layer chromatography analysis shows that the Ghanaian (Mu1059) and Malaysian (Mu1615) strain produce mycolactone, but the Australian type strain (Mu1617) does not.
(4.46 MB TIF)
