# Supplementary material for: BCG-Mediated Protection against Mycobacterium ulcerans Infection in the Mouse
Source: PLoS Negl Trop Dis. 2011 Mar 15;5(3):e985. doi: 10.1371/journal.pntd.0000985 (PMC3057947; doi:10.1371/journal.pntd.0000985)
Supplement: Figure S1 — Proinflammatory, Th1, and Th2 cytokine production after BCG vaccination before and after M. ulcerans challenge. (0.04 MB DOC) [file pntd.0000985.s001.doc]

Figure S1

Proinflammatory cytokines:

Th1 cytokines:

Th2 cytokines:
