# Supplementary material for: BCG-Mediated Protection against Mycobacterium ulcerans Infection in the Mouse
Source: PLoS Negl Trop Dis. 2011 Mar 15;5(3):e985. doi: 10.1371/journal.pntd.0000985 (PMC3057947; doi:10.1371/journal.pntd.0000985)
Supplement: Figure S2 — Chemokine production after BCG vaccination before and after M. ulcerans challenge. (0.03 MB DOC) [file pntd.0000985.s002.doc]

Figure S2

Chemokines:
