# Supplementary figures and images for: BCG-Mediated Protection against Mycobacterium ulcerans Infection in the Mouse
Source: PLoS Negl Trop Dis. 2011 Mar 15;5(3):e985. doi: 10.1371/journal.pntd.0000985 (PMC3057947; doi:10.1371/journal.pntd.0000985)

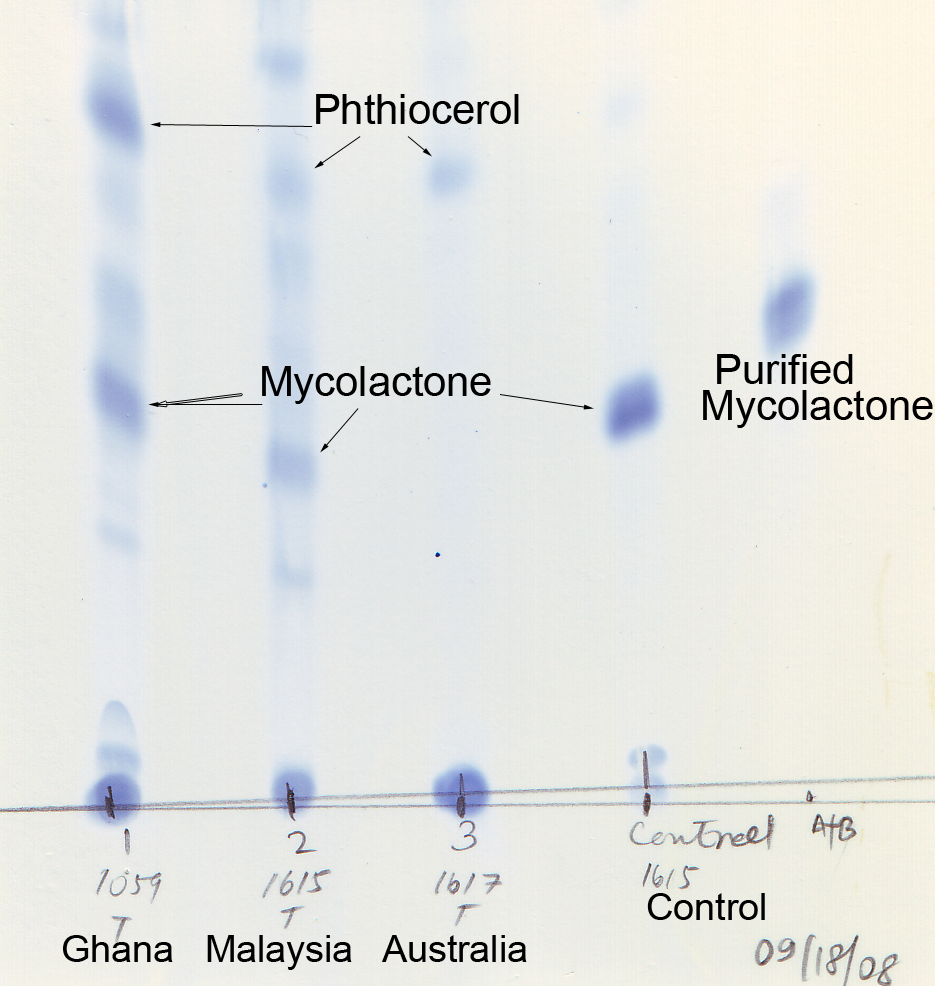

Supplement: Figure S3 — Thin layer chromatography analysis shows that the Ghanaian (Mu1059) and Malaysian (Mu1615) strain produce mycolactone, but the Australian type strain (Mu1617) does not. (4.46 MB TIF) [file pntd.0000985.s003.tif]
